# Methadone Withdrawal-Induced Psychosis: A Case Report

**DOI:** 10.7759/cureus.55256

**Published:** 2024-02-29

**Authors:** Oluwaseun Oke, Marta B Sekh, Bamidele O Johnson, Samuel Adeyemo

**Affiliations:** 1 Department of Psychiatry, Interfaith Medical Center, Brooklyn, USA; 2 Department of Medicine, American University of Antigua, Osbourn, ATG

**Keywords:** withdrawal, hallucination, delusion, opioid, methadone

## Abstract

Methadone is a synthetic full µ-opioid receptor agonist and N-methyl-D-aspartate antagonist given to patients who have recently stopped using illicit opioids or are tapering off chronic opioid pain medication. Maintenance treatment with methadone is today the most widespread and effective way to treat opiate addiction, which achieves abstinence, decreases morbidity and mortality, improves quality of life, and reduces crime genesis, among other benefits. It is also approved by the Food and Drug Administration for treating moderate-to-severe pain that remains unresponsive to nonopioid medications. Patients sometimes abruptly discontinue the medication for several reasons and sometimes suffer distressing but non-life-threatening withdrawal symptoms. More common withdrawal symptoms include anxiety, agitation, rhinorrhea, nausea, and vomiting, like other opioid agonist medications. Psychosis has been reported in some rare cases of methadone withdrawal. However, more research is required because, although psychotic symptoms have been described in different case reports after the reduction or withdrawal of methadone, they have not been sufficient. This case report contributes to the literature on rare manifestations of psychosis in patients who abruptly discontinue the use of methadone.

## Introduction

Methadone is the gold standard for Food and Drug Administration-approved medication-assisted therapy (MAT) for opioid use disorder in the United States. Buprenorphine and naltrexone are the other two medications that are currently registered and approved for MAT. However, research has shown that oral methadone is more effective and allows for a longer duration of treatment, which is associated with better outcomes [1]. Methadone is a synthetic full µ-opioid receptor agonist and N-methyl-D-aspartate antagonist first introduced for MAT in the 1960s [2]. It is used as a form of agonist therapy in patients who have recently discontinued the use of illicit opioids or are tapering off chronic opioid pain medication. Methadone has a strong affinity for opioid receptors. When bound, it reduces the cravings and symptoms of opioid withdrawal without inducing euphoria, which helps treat opioid use disorder and prevents relapse. Methadone maintenance therapy is often long-term to improve remission and requires tapering prior to discontinuation. Once the individual has decided to come off the medication, the dose is tapered gradually by a 5-10% reduction weekly, biweekly, or monthly while monitoring for any withdrawal symptoms, usually in the outpatient setting. More rapid tapering could be performed, but it requires a controlled setting with non-opioid medications to provide symptomatic relief for methadone withdrawal symptoms. There are no set methadone reduction protocols, and they must be tailored to each patient’s physiological response [3-5].

Methadone withdrawal symptoms are usually not life-threatening but can cause much distress to the individual. Common signs and symptoms of methadone withdrawal result from central nervous system arousal and sympathetic hyperactivity, which include anxiety, insomnia, agitation, mydriasis, yawning, lacrimation, muscle cramps, tachycardia, and hypertension; flu-like symptoms such as rhinorrhea, diaphoresis, piloerection, myalgia, and arthralgia; as well as gastrointestinal symptoms such as nausea, vomiting, diarrhea, and abdominal pain, among others. The withdrawal symptoms may vary depending on the amount of the drug used and the day of the withdrawal from the initial onset of symptoms [6-8]. In the world of narcotics, some individuals say that they would much rather withdraw from heroin without any medical assistance than undergo short-term methadone-assisted detoxification [9]. Since methadone is a long-acting opioid agonist, the individual usually does not develop acute withdrawal symptoms. However, there have been reports of withdrawal onset as early as three days after interruption of the maintenance dose and lasting more than two weeks. Patients with severe methadone use disorder report more severe withdrawal symptoms than patients with severe heroin use disorder when undergoing a gradual methadone reduction program to wean off the opioid completely [8]. Physiological withdrawal symptoms are common and can be controlled with other non-opioid medications, such as clonidine [10]. However, there have been reports of rare psychological withdrawal symptoms that are more difficult to anticipate.

Psychological symptoms of methadone withdrawal include dysphoria, depression, suicide ideation, and the manifestation of psychosis [7,11]. The atypical depression due to methadone withdrawal is characterized by insomnia, fatigue, anxiety, and hypochondriasis [12]. Psychotic symptoms reported include paranoid delusions and auditory, visual, and acoustic hallucinations. This case report contributes to expanding the body of literature available on rare presentations of methadone withdrawal psychosis.

## Case presentation

Case summary

Ms. JS, a 69-year-old African American female with a collateral-reported psychiatric history of depression and a past medical history of opioid (heroin) use disorder, diabetes mellitus, hypertension, and seizure disorder, was brought into the emergency department for worsening paranoia of one-week duration.

On evaluation, the patient was hypervigilant, looking all around the room, and suspicious. The patient reported, "I am paranoid and feel like I am being followed, maybe because I am getting off methadone." The patient reported she had been unable to sleep for a few days and took Benadryl. The patient denied any auditory or visual hallucinations. She reported feeling anxious but denied depressive or manic symptoms.

The patient endorsed a 20-year history of heroin use and reported that her last use was about two years prior to hospital presentation, which was when she initially started an outpatient methadone maintenance treatment program (MMTP). She reported using 70 mg of methadone per oral daily, which had been tapered to 30 mg per oral daily. The patient reported getting tired of using methadone and therefore stopped going to the MMTP. She reported a personal decision to quit using methadone, as she doesn't want to use it indefinitely. Per the patient's MMTP, the patient picked up and took her methadone daily. However, she last picked up methadone nine days prior to the hospital presentation. She also reported social alcohol intake. She denied any other substance use.

On the mental status examination, the patient appeared disheveled with poor grooming. She was guarded and uncooperative. Her effect was irritable, constricted, and reactive. Her thought process was circumstantial. She endorsed paranoid delusions. She denied suicidal and homicidal ideations, intent, or plans, and denied auditory or visual hallucinations. Her insight, judgment, and impulse control were limited. Her Folstein mini-mental status exam score was within the normal range.

Collateral was obtained from the patient's daughter. She reported that the patient became verbally abusive and aggressive with her because she did not give her Benadryl. The daughter reported that the patient had become confused and very distracted about one week prior to the hospital presentation. She also reported that the patient went to a store and claimed a man was taking photos of her and that people were looking at her through their windows. She accused her daughter of speaking about her and setting her up when they communicated over the phone three days prior. The patient’s daughter stated that the patient’s only psychiatric history and hospitalization were 19 years prior. At that time, the patient was depressed in the context of bereavement and was found running in the streets naked. She has not received any other psychiatric follow-up except for treatment of opioid use disorder.

Investigations

The complete blood count (Table 1), complete metabolic profile, thyroid function tests (TSH and free T4), and vitamin B12 (Table 2) were within normal limits. Urinalysis was normal. Urine toxicology was positive for methadone (Table 3).

**Table 1:** Values of the complete blood count

Complete blood count (CBC)	Result	Normal range
White blood cell (WBC)	8.4	4.5-10.2 10x3/ul
Red blood cell (RBC)	5.01	3.95-4.83 10x6/ul
Hemoglobin (HBG)	14.5	11.4-15.5 g/dl
Hematocrit (HCT)	43.6	37-43.7%
Mean corpuscular volume (MCV)	87.1	82-94.5 fl
Mean corpuscular hemoglobin (MCH)	28.8	26.9-32.5 pg
Mean corpuscular hemoglobin concentration (MCHC)	33.1	31.9-35.0 g/dl
Red cell distribution width (RDW)	16.9	12.6-14.9%
Platelets	223	180-401 10x3/ul
Mean platelet volume (MPV)	8.1	7.9-10.6 fl
Neutrophils auto	59	47.9-74.1%
Lymphocytes auto	33	17.8-41.1%
Monocytes auto	6.4	4.5-10.7%
Eosinophils auto	0.5	0.1-4.7%
Basophils auto	1.1	0.3-1.1%
Neutrophils absolute	5.0	2.3-6.8 10x3/ul
Lymphocytes absolute	2.8	1.3-3.0 10x3/ul
Monocytes absolute	0.5	0.3-0.9 10x3/ul
Eosinophils absolute	0.0	0.0-0.5 10x3/ul
Basophils absolute	0.1	0.0-0.1 10x3/ul

**Table 2:** Values of the complete metabolic profile, thyroid stimulating hormone (TSH), free T4, and vitamin B12

Complete metabolic profile (CMP)	Result	Normal range
Phosphorus	3.8	2.5-4.5 mg/dl
Magnesium	2	1.6-2.3 mg/dl
Bilirubin total	0.8	0.2-1.3 mg/dl
Bilirubin, direct	0.4	0.0-0.4 mg/dl
Aspartate aminotransferase (AST)	20	14-36 U/L
Alanine aminotransferase (ALT)	16	< 35 U/L
Protein, total	7.6	6.3-8.2 g/dl
Albumin	4.4	3.5-5.0 g/dl
Alkaline phosphatase	96	38.0-126.0 U/L
Vitamin B12	359	239-931 pg/ml
Thyroid-stimulating hormone (TSH)	2.41	0.465-4.680 uIU/ml
Thyroxine (free T4)	1.1	0.78-2.19 ng/dl
Glucose	116	70-99 mg/dl
Blood urea nitrogen	12	7.0-17.0 mg/dl
Creatinine	1.03	0.52-1.04 mmol/L
Sodium	142	133-145 mmol/L
Potassium	3.5	3.5-5.1 mmol/L
Chloride	105	98-107 mmol/L
Carbon dioxide (CO2)	30	22-30 mmol/L
Calcium	10	8.4-10.5 mg/dl
Anion gap	7	7-17 mmol/L
Estimated glomerular filtration rate (eGFR)	58.9	≥90.0 mL/min/1.73 m2

**Table 3:** Result of urine toxicology

Urine toxicology	Result
Cocaine screen	Negative
Opiate screen	Negative
Phencyclidine (PCP) screen	Negative
Cannabinoid screen	Negative
Barbiturate screen	Negative
Benzodiazepine screen	Negative
Amphetamines screen	Negative
Methadone screen	Positive

**Figure 1:**
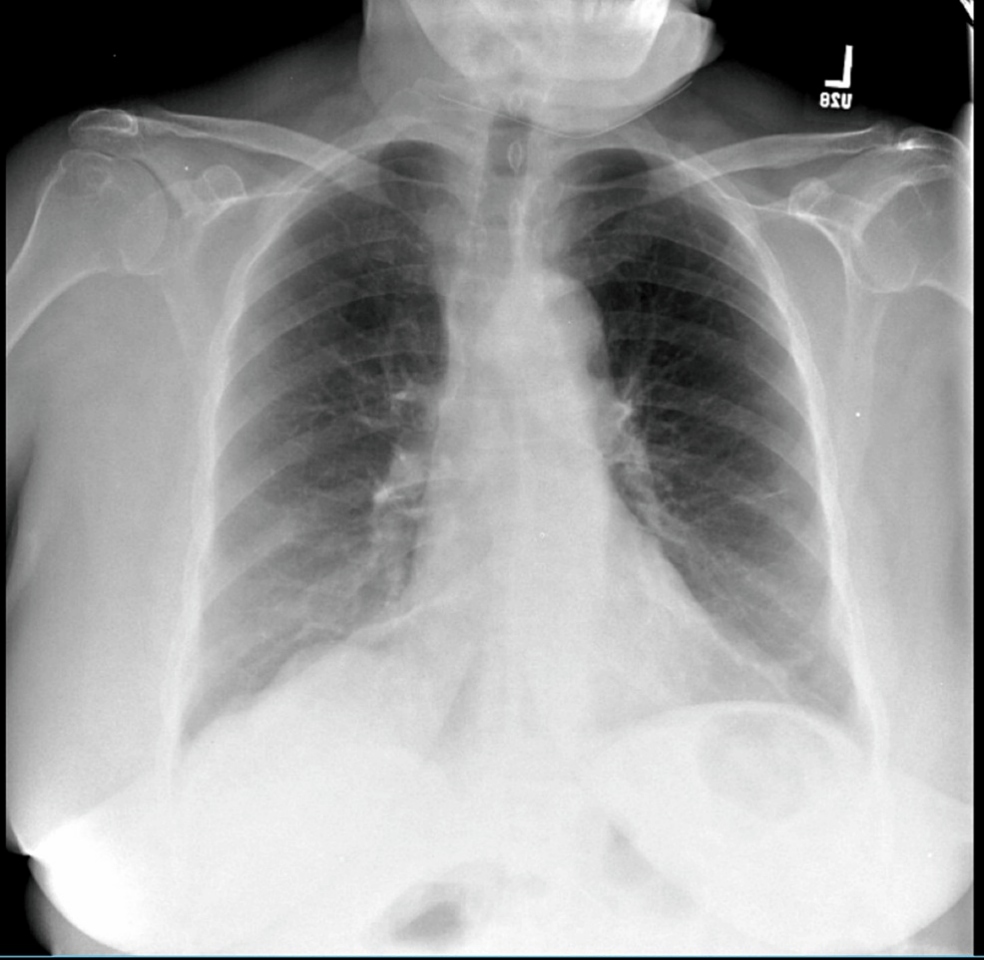
Chest X-ray showing mild atelectasis in both lung bases

The chest X-ray showed mild atelectasis in both lung bases (Figure 1). A CT scan showed no evidence of intracranial mass, hemorrhage, infarct, hydrocephalus, skull fracture, or extra-axial fluid collection.

Differential diagnoses

The possibility of psychosis due to a general medical condition was considered due to the presentation of paranoid delusions alongside a history of diabetes mellitus and hypertension. However, this was ruled out after there was no significant identifiable evidence from the patient’s history, collateral history, physical examination, laboratory investigation, or imaging results that the patient's condition is a direct physiological consequence of the patient's medical conditions.

A second possibility of delirium was considered due to the patient's presentation of psychosis following substance withdrawal as well as a collateral history of confusion earlier in the week. However, this was ruled out as there was no observable disturbance in attention or cognition upon evaluation. The patient was fully alert and oriented in time, place, and person. Folstein's mini-mental status exam score was within the normal range.

Another differential diagnosis considered is other substance- or medication-induced psychosis. However, this was ruled out after there was no significant identifiable evidence from the patient's history or urine toxicology results of the patient using other substances or medications. Urine toxicology was only positive for methadone.

Treatment

The patient was started on aripiprazole 2 mg, which was increased to 5 mg orally daily for psychosis. She was also started on hydroxyzine 25 mg three times daily as needed (PRN) for anxiety. From day 1 to day 17, the patient continued to decline the resumption of methadone use. She continued to endorse paranoid ideations and unresolving anxiety symptoms despite PRN anxiety medication and antipsychotics. By day 18, she agreed to be restarted on methadone 10 mg daily, and hydroxyzine was discontinued. She began reporting improvements in anxiety by day 20 and eventually stopped endorsing paranoid ideations.

Outcome and follow-up

The patient remained stable on the unit and, by day 22, was discharged. Ms. JS continued aripiprazole 5 mg daily and was scheduled to follow up in an outpatient psychiatric treatment and substance use treatment program. At a three-month follow-up by the case manager, the patient continued to visit her MMTP for her daily methadone and reported no further symptoms of psychosis or anxiety.

## Discussion

Psychosis is an uncommon presentation of methadone withdrawal. As with methadone, psychosis has also been reported in the withdrawal phase of other opioids such as buprenorphine [13], morphine [14], tramadol [15], and oxycodone [16]. Psychotic presentations vary by different case reports available, but commonly reported include auditory, visual, and acoustic hallucinations, delusional ideations, and paranoid delusions [17-19]. The severity of the psychotic presentations also varies depending on the different cases reported. However, it is unclear what factors determine the severity of psychotic symptoms. Our patient only endorsed paranoid delusions.

Lozano et al. reported seven cases of psychosis after the patient stopped taking methadone [11]. The time factor is a common denominator among the cases reported. Psychotic symptoms appeared days after stopping methadone and disappeared after the resumption of methadone, as with Ms. JS. Also, two cases reported psychosis triggered by the voluntary slow-dose reduction of the long-term methadone maintenance treatment. Individuals in these cases responded well to the treatment with an increased methadone dose [17].

It is currently unclear the exact mechanism by which methadone withdrawal results in psychosis. However, it has been observed in basic experiments that opioid agonists alter dopaminergic activity, including metabolism, uptake, and release in the substantia nigra and striatum [20]. This mechanism may also explain why methadone has been used as an adjunct treatment for psychosis in patients with resistant schizophrenia with marked improvement. Furthermore, its use has been reported to be beneficial in reducing the dosage required in patients being treated with antipsychotics [11]. With this association, withdrawal from methadone could possibly predispose patients to episodes of psychosis [11].

## Conclusions

Patients on long-term treatment for opioid use could occasionally get impatient and frustrated because of the length of time it takes for their treatment and therefore become non-compliant. Also important are the associated side effects of the medications and the stigma associated with going to a drug treatment program. It is essential for patients to be educated on the importance of methadone compliance and the adverse effects of abruptly stopping the medication, even at low doses. This case report contributes to the body of knowledge available, showing that psychosis can occur when abruptly stopping methadone and symptoms can resolve soon after a low dose of methadone is reintroduced.

As clinicians, it is imperative to pay attention to other causes of psychosis, such as methadone withdrawal, particularly among patients presenting with a primary psychotic disorder. Additionally, early recognition and treatment of psychosis is of utmost importance among patients on methadone maintenance treatment without a history of primary psychotic disorder who may or may not present with other symptoms of methadone withdrawal.
